# Effectiveness of neutral electrolyzed water and copper oxychloride on fungi spores isolated from tropical fruits

**DOI:** 10.1016/j.heliyon.2021.e07935

**Published:** 2021-09-07

**Authors:** Alfonso Vásquez-López, Rafael Gómez-Jaimes, Tania Villarreal-Barajas

**Affiliations:** a Instituto Politécnico Nacional, CIIDIR Unidad Oaxaca, Santa Cruz Xoxocotlán, 71230, Oaxaca, Mexico; b Instituto Nacional de Investigaciones Forestales, Agrícolas y Pecuarias, Santiago Ixcuintla, Nayarit, Mexico; c Esteripharma SA de CV, Atlacomulco, 50450, State of Mexico, Mexico

**Keywords:** Disinfection, Fungi, Neutral electrolyzed water, Post-harvest, Tropical fruits

## Abstract

The aim of this study was to assess the antifungal effectiveness of neutral electrolyzed water (NEW) to inhibit the spore germination of post-harvest fungi common in fruits, determine the required available chlorine concentration (ACC) of NEW and to compare it with copper oxychloride (CO) and sterile distilled water (SDW) *in vitro*. This study evaluated the biological effectiveness of NEW to inactivate pure cultures of 11 different fungi obtained from post-harvest tropical fruits with anthracnose, rottenness or necrosis symptoms. A conidial solution of 1 × 10^4^ spores/mL per culture was prepared and treated with a low, medium and high ACC of NEW (pH 7.0 ± 0.05, 12, 33 and 53 mg/L of ACC and ORP of 850 mV), CO at 0.3 g/L, or sterile distilled water as a control, for 3-, 5- and 10-min contact time. Spore germination of *Alternaria alternata*, *Botrytis cinerea*, *Cladosporium australiense, Colletotrichum gloeosporioides* and *C. siamense*, *Fusarium solani* and *F. oxysporum*, and *Lasiodiplodia theobromae* was inhibited in 100% by NEW at 12, 33 and 53 ppm ACC; 3,5 and 10 min contact time. *Aspergillus niger* and *A. tamarii* required 53 mg/L ACC to inhibit 100% of spore germination. NEW at 33 and 12 mg/L inhibited around 50% and <25% of *A. niger* spore germination, respectively. NEW at 53 mg/L ACC was the most efficient treatment against *Rhizopus stolonifer* but only inhibited spore germination in ∼25%. CO inhibited spore germination by 100% of *A. alternata*, *B. cinerea*, *C. australiense, C. gloeosporioides, C. siamense* and *L. theobromae*. However, CO inhibited <25% of spore germination of *F. solani, F. oxysporum, A. niger*, *A. tamarii* and *R. stolonifer.* NEW and CO had a significant effect on every fungus compared to a SDW treatment. SDW was the least effective treatment, followed by CO. NEW at 12 mg/L and 33 mg/L ACC were equally effective in eliminating the fungi, and more effective than CO. NEW at a concentration of 53 mg/L ACC was the most effective treatment. Results obtained in this study show that NEW has effectively inhibited spore germination of these species, and this treatment could be used as a substitute an ecological novel alternative to CO to avoid spore growth in the above-mentioned fruits.

## 1 Introduction

The south-southeast region of Mexico, which includes the states of Campeche, Chiapas, Guerrero, Oaxaca, Puebla, Quintana Roo, Tabasco, Veracruz and Yucatán, is of great relevance in the agricultural sector; the region contributes approximately 60 and 5% of the national production of fruits and vegetables, respectively (SADER (Secretaría de Agricultura y Desarrollo Rural) et al., 2018). However, approximately 25% of this production is lost in the post-harvest stage because of microorganisms, mainly fungi (SADER (Secretaría de Agricultura y Desarrollo Rural) et al., 2018). For prevention of postharvest diseases of fruits and vegetables, there are three main methods such as physical, chemical and biological. Regarding chemical sanitizers, nowadays farmers are using mainly (a) sodium and calcium hypochlorite, (b) plant-based antimicrobial formulations, (c) peracetic acid, (d) fungicides and (e) hydrogen peroxide, (personal research, Siddiqui, 2018). While chlorine remains the most widely used chemical sanitizer in the fresh produce industry, there is a trend to reduce the use of this disinfectant as it can lead to the formation of organochlorinated carcinogenic compounds. Plant-based antimicrobial formulations such as citric extracts are well accepted among the farmers and some examples of commercial plant-based disinfectants are Citrik Agro, Citripower 1000 and G-Citrox. Most of them are not harmful to the environment and human beings but only in the recommended dilutions and even protection equipment is required during the application. Peracetic acid is equally effective than chlorine compounds, but shows pungent odor in confined areas, it has irritant vapors during inhalation and it is considered as a hazardous oxidizer on skin. Fungicides such as copper oxychloride, thiabendazole, azoxystrobin, and difenoconazole, among others, are effective but with time, fungi become resistant and fungicides are well known to be environmental un-friendly and leave residues. Hydrogen peroxide (H_2_O_2_) is an alternative sanitizing agent recognized by FDA as safe but it was observed that it causes phytotoxicity in the both the stem and the fruit (Sehirli et al., 2020). A viable alternative is the use of Electrolyzed Water (EW) which has gained more interest recently in food and surfaces disinfection. The collection of papers, reviews and books focused on postharvest EW treatment indicated that there is great potential for successful implementation by industry because it has strong efficacy capabilities, does not produce harmful by-products or leave behind residues, and is accepted for use in organic production (Villarreal-Barajas et al., 2022). It is considered not toxic to public health not needing protection equipment, it is easy and safe to preparate and handle, no odor or irritant vapors are released even at a concentrated form, it does not cause phytotoxicity, it is eco-friendly and is commercially feasible either producing it *in situ* with low cost of production or obtaining it from a supplier site.

EW can be produced as acid EW (Buck et al., 2002) (pH around 2–4), near-NEW (Guentzel et al., 2010) (pH around 5.5–6.5), neutral electrolyzed water (NEW) (Vásquez-López et al., 2016) (pH around 6.5–7.5) and alkaline EW (Youssef and Hussien, 2020) (pH around 8–11). The antimicrobial mechanism of all four waters depend mainly on three physicochemical properties: pH, oxidation-reduction potential (ORP) and available chlorine concentration (ACC). NEW has several advantages such as a) greater stability of chlorine agents and safe for consumers' health (Hamidi et al., 2020) since NEW can be reverted back to water and salt without producing an excessive release of toxic substances (Ramírez Orejel and Cano-Buendía, 2020), b) potential for reducing pesticide residues on fresh produce (Han et al., 2017; Hu et al., 2016; Qi et al., 2018) c) no residues of NEW on the fresh produce after its application (Rahman and Murshed, 2019) d) no cross contamination resulting from handling, transportation and packaging (Afari et al., 2015) and c) it has been reported that NEW does not have negative effects on the physical, organoleptic, nutritional qualities and chemical components of fresh produce (Aday, 2016; Hao et al., 2015). However, the EW main disadvantage of EW is that its effectiveness is reduced in the presence of organic compounds weakening its antimicrobial capacity (Duan et al., 2016; Zhang et al., 2016). NEW is made by the electrolysis of dissolved salt; sodium chloride (NaCl) in deionized water to generate mainly hypochlorous acid (HOCl; 95%) and other chlorine forms such as hypochlorite ions and trace amounts of chlorine (Cl_2_; Guentzel et al., 2008). Hypochlorous acid in EW is 80 times more effective than the hypochlorite ion (Kim et al., 2000). NEW (pH 7, ORP 800–900 mV, and 10–200 mg/L ACC) does not lose chlorine as fast as acid EW (Guentzel et al., 2010), and thus presents less health problems and reduces phytotoxic secondary effects.

Although there are many studies focusing on the antibacterial effects of NEW, research focusing on the antifungal effects of NEW is scarce. Some studies have demonstrated promising effects of NEW on *Botrytis cinerea* and *Monilia fructicola* (isolation origin not indicated; Guentzel et al., 2010), on *Aspergillus* spores isolated from peanuts seeds (Xiong et al., 2010), and on *Fusarium* isolated from cereals (Audenaert et al., 2012). Very few studies however, have documented the potential of NEW on diverse fungi isolated from tropical fruits. Consequently, the aim of this study was to evaluate the effectiveness of NEW on fungi spores isolated from tropical fruits, determine the required ACC of NEW and to compare it with CO.

## 2 Materials and methods

### 2.1 Neutral electrolyzed water

The NEW used in this study was produced by electrolysis of a continuous supply of room temperature saturated NaCl solution diluted in water using two patented generators (Esteripharma S.A. de C.V., State of Mexico, Mexico). The resulting NEW with neutral pH (7.0 ± 0.05) and an ORP of 850 mV, was used at 12, 33 and 53 mg/L of ACC, which was verified using a chlorine ultra-high range ISM meter (model HI 96771C, Hanna Instruments, Melrose, MA).

### 2.2 Fungal isolates

Samples of ripened fruit; banana (*Musa paradisiaca* L.), canario chili (*Capsicum pubescens* Ruiz & Pav), habanero chili (*Capsicum chinense* Jacq.), papaya (*Carica papaya* L.), pineapple (*Ananas comosus* (L.) Merr), strawberry (*Fragaria x ananassa* (Weston) Duchesne), tomato (*Solanum lycopersicum* L.) and peach (*Prunus persica* (L). Batsch) were obtained from crop fields around the southeast region of Mexico (Oaxaca, Chiapas, Tabasco and Veracruz) in August and September 2015. Fruits collected in Chiapas were processed in the *Instituto Nacional de Investigaciones Forestales, Agrícolas y Pecuarias (INIFAP)*, Chiapas, Mexico; fruits collected in Tabasco were processed in the *Universidad Autónoma Chapingo*, Tabasco, Mexico; and fruits collected in Oaxaca and Veracruz were processed in the *Centro Interdisciplinario de Investigación para el Desarrollo Integral Regional (CIIDIR Oaxaca)* of the *Instituto Politécnico Nacional*, Mexico. Fruit samples were transported in refrigerated conditions to each laboratory and stored in an ice box for further analyses the same day. From each fruit, random samples were chosen, washed with soap and distilled water and dried on sterile paper in a laminar hood. Rotten samples of 0.5 cm^2^ from each fruit were removed, washed with sodium hypochlorite at 1.0% for 3 min and rinsed three times with distilled water. The samples were cultured in potato dextrose agar (PDA; BD Bioxon®, Mexico City, Mexico) petri dishes, stored at 25 °C and exposed to 8 h light and 16 h darkness for three days. The fruit pieces were observed daily to evaluate the presence mycelial growth. Different mycelia were subcultured to obtain pure isolated fungi cultures. Each isolated fungus was preserved in tubes with potato dextrose agar (PDA) covered with mineral sterile oil for morphological and molecular identification. Preliminary morphological identification of the fungi was carried out with Barnett and Hunter (Barnett and Hunter, 1998) taxonomic key. Molecular identification was performed in the Phytosanitary Diagnostic Laboratory of the *Colegio de Postgraduados*, State of Mexico, Mexico using the (Ahrens and Seemüller, 1992) methodology. The fungal ITS1 and ITS2 regions were amplified using universal primers ITS5 (GGA AGT AAA AGT CGT AAC AAG G) and ITS4 (TCC TCC GCT TAT TGA TAT GC) (White et al., 1990). The amplified product was purified with the Wizard® Genomic DNA Purification Kit (Promega Corporation) and sequenced with the ABI PRISM® 3100 Genetic Analyzer (Hitachi, Ltd.) from Applied Biosystem. Sequences were analyzed with SeqManII, LaserGene software of DNASTAR (DNAStar Inc., USA) and aligned with available sequences in GenBank at the National Center for Biotechnological Information (NCBI) (www.ncbi.nlm.nih.gov). The sequences with greatest similarity were compared with our sequences. Eleven fungi were isolated from fruits with rottenness, anthracnose and necrosis symptoms (Table 1).

**Table 1:** Morphological and molecular identification of fungal isolates.

Fruit	Fungal isolate	Morphological identification	Molecular identification	
		According to the following author	Access number (base pair)	NCBI % homologation
Pineapple	*Alternaria alternata*	(Guevara et al., 2019)	MH560609 (501)	100%
Tomato	*Aspergillus niger*	(Xu et al., 2015)	HQ850370.1 (591)	99.83%
Tomato	*Botrytis cinerea*	(Li and Schnabel, 2011)	MN589850 (569)	100%
Tomato	*Cladosporium australiense*	(Chen et al., 2009)	MN341230.1 (549)	100%
Banana	*Colletotrichum gloeosporioides*	(Riera et al., 2019)	MH865167.1 (577)	99.83%
Papaya	*Colletotrichum siamense*	(Chung et al., 2019)	MT434615.1 (593)	100 %
Chili canario	*Fusarium oxysporum*	(Moya-Elizondo et al., 2019)	GU724514.1 (539)	99.07%
Chili habanero	*Fusarium solani*	(Ramteke et al., 2020)	KR708647.1 (550)	100%
Banana	*Lasiodiplodia theobromae*	(Serrato-Diaz et al., 2013)	EU918707.1 (566)	100%
Strawberry	*Rhizopus stolonifer*	(García-Estrada et al., 2019)	AB113022.1 (852)	89.01%
Peach	*Aspergillus tamarii*	∗	MK638758.1 (1747)	100%

∗ *A. tamarii* was identified only molecularly.

### 2.3 Effect of NEW and CO on spore germination of different fungi in pure culture

Individual cultures of each fungus were grown separately in PDA petri dishes, stored at 25 °C and exposed to 12 h light and 12 h darkness for 10–20 days to obtain only asexual cells. The conidia were removed from the plate surface with a sterilized angled glass rod. A conidial suspension was obtained by adding 2 mL of sterile distilled water (SDW) followed by the filtering through three layers of sterile gauze to remove mycelia. Serial dilutions were made by taking 100 μL of the filtered solution and adding 10 mL of SDW to reach a concentration of 10^4^ spores/mL. The conidial density of each fungus was measured in a Neubauer chamber (Marienfeld, Germany). A volume of 1.0 mL of each fungus (10^4^ spores/mL) was combined with 9.0 mL of NEW at a low, medium and high ACC of NEW (12, 33 or 53 mg/L ACC). Control treatments consisted of 1.0 mL of each fungus and 9.0 mL of SDW. A copper oxychloride (CO) treatment was included in the experiment with a concentration of 0.3 g/L and served as a positive fungicidal control. Each pathogenic fungus sample was tested five times with every NEW concentration, with SDW or with CO. Each treatment consisted of 5 repetitions. The treatments were vortexed and incubated at 25 °C for 3, 5 and 10 min. Following the treatments, 20 μL of each treatment were plated individually in five equidistant points of the Petri dish on the PDA. Plates were sealed and incubated at 25 °C, 12 h light and 12 h darkness for 24 h. To assess the effectiveness of CO and NEW on the inhibition rate of each fungus, the spore germination was enumerated counting microscopically the germination of 100 spores on each of the 5 plated points.

### 2.4 Statistical analyses

The percentage of inhibition of conidia germination was estimated with the formula: I= (C-T/C) ∗100. Where: I = percentage of non-germinated conidia; C = number of germinated conidia in the control treatment at time *i*; T = number of germinated conidia in the experimental treatment at time *i*. Counts of spore germination from all treatments were transformed using Log_10_(x+1). The model with the best set of explanatory variables was obtained using a backward step procedure starting with the full model (fungi species, time of contact, treatment and repetitions) which proceeded to test the inclusion of each variable one by one (Zuur et al., 2009). Analysis of variance (ANOVA) and Tukey HSD analysis (where differences were found) were used to compare family-wise means of treatments significance across all species, and for each species separately to analyze statistical differences. Data were analyzed using statistical analysis software (MS-Excel 2019, Microsoft). Statistical analyses were performed using R (R Core Team, 2021) and differences were considered significant at p < 0.05.

## 3 Results and discussion

### 3.1 Effect of NEW and CO on spore germination of different fungi in pure culture

**Figure 1:**
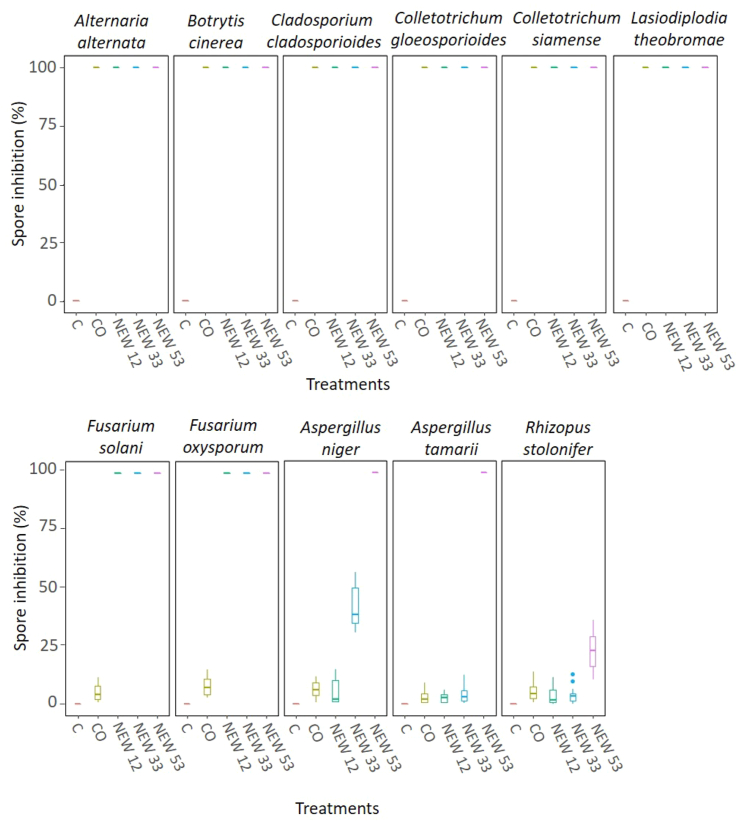
Box plots indicate the spore inhibition percentage (%) of each fungus for each treatment: control (sterile distilled water), copper oxychloride (CO) and neutral electrolyzed water (NEW) at 12, 22 and 53 mg/L available chlorine content (ACC). Lines within each box represent medians and the whiskers extend to the most extreme data which is no further than 2 standard deviations. Data beyond whiskers is shown as blue dots. We present the compilation of data from each treatment at three different time samples and five replicates each, (n = 15 observations in each box).

**Figure 2:**
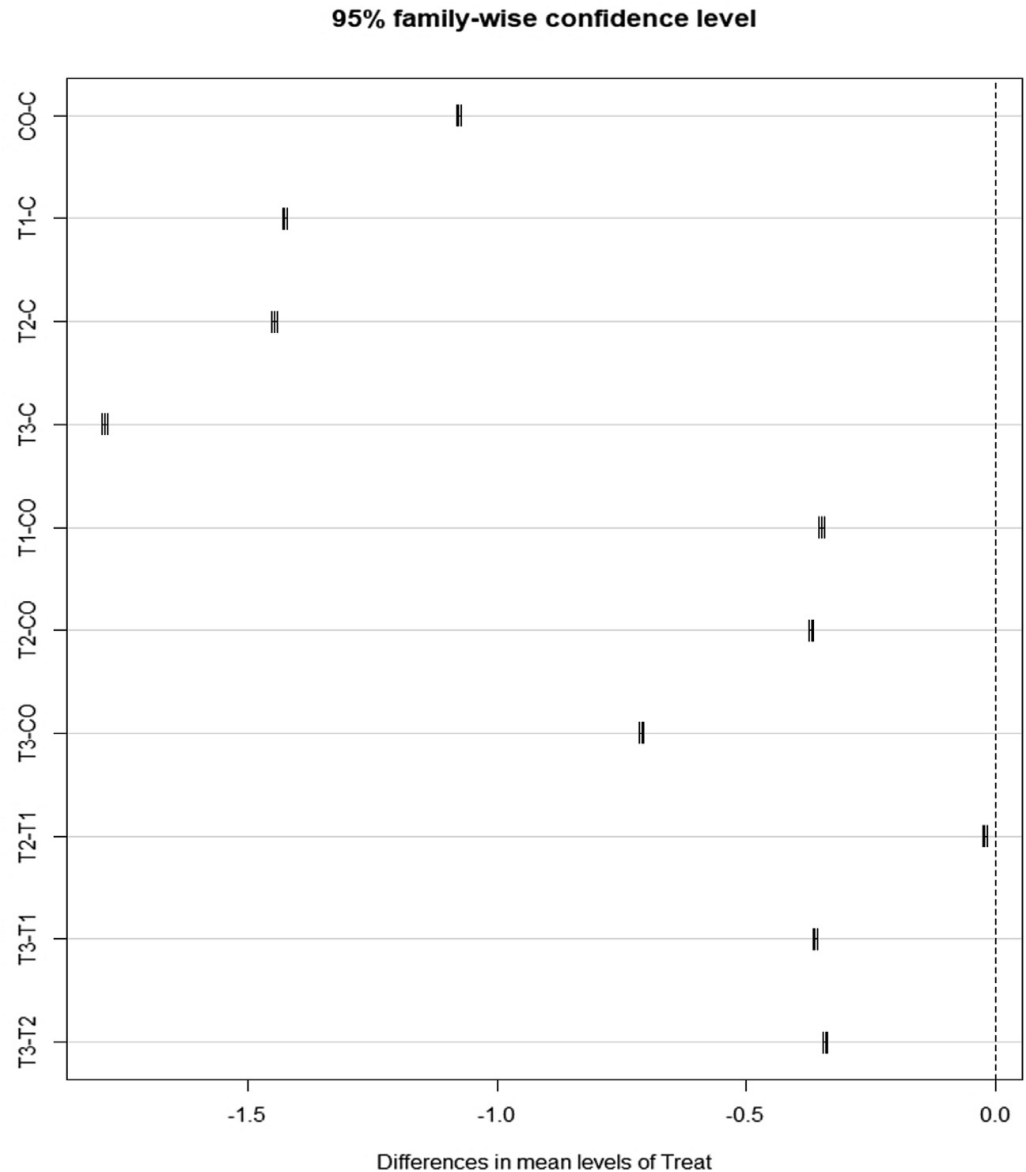
Family-wise Tukey HSD plot showing differences across treatments. (C) Control (sterile distilled water), (CO) copper oxychloride and neutral electrolyzed water (NEW) at (T1) 12, (T2) 22 and (T3) 53 mg/L available chlorine content (ACC).

The effectiveness of NEW and CO to inactivate fungi in pure culture has been assessed using eleven organisms. These organisms are common postharvest fruit pathogens reported to be present on pineapple, banana, papaya, grapes, strawberries, peaches, tomato and chili, fruit processing equipment, and in food storage areas (Al-Haq et al., 2001). All treatments had a significant effect on all species when compared to the controls. NEW, starting at a low ACC as 12 mg/L ACC, and 3-, 5- and 10-min contact time, inhibited 100% the conidia germination of *A. alternata, B. cinerea, C. australiense*, *C. gloeosporioides*, *C. siamense*, *F. solani, F. oxysporum* and *L. theobromae* compared to SDW (p < 0.0001). In contrast, CO was 100% effective to inhibit conidia growth of *A. alternata*, *B. cinerea*, *C. australiense, C. gloeosporioides, C. siamense* and *L. theobromae* (Figure 1). Low and medium concentrations of NEW (12 mg/L and 33 mg/L ACC) were equally effective in eliminating the fungi, and more effective than CO. However, CO was the least effective treatment after SDW (control) and NEW at a high concentration (53 mg/L ACC) was the most effective treatment (Figure 2). NEW at a high concentration (53 mg/L ACC) inhibit 100% *A. niger* and *A. tamarii* spore germination but only inhibited spore germination in ∼25% against *Rhizopus stolonifer*. CO inhibited <25% of spore germination of *F. solani, F. oxysporum, A. niger*, *A. tamarii* and *R. stolonifer.*

### 3.2 Effect of low ACC of NEW and CO on spore germination of different fungi in pure culture

*A. alternata* (isolated from pineapple) and *C. australiense* (isolated from tomato) spore germination was inhibited 100% by NEW at 12 mg/L ACC and 3 min of treatment and by CO at 0.3 g/L. Other studies have also observed complete inactivation of fungi in pure culture using acid EW (pH = 2.8), a high concentration of ACC (54 mg/L) and less contact time (30 s). Buck et al. (2002) reported that acid EW eliminated *Alternaria spp., Aspergillus flavus* and *A. niger, Botrytis sp., Colletotrichum sp., Cladosporium sp. Fusarium moliniforme* and *Fusarium sp.* isolated from various symptomatic plant samples in pure culture. Acid EW has antifungal properties, but quickly lose chlorine gas, thus reducing the effectiveness of the active ingredient. NEW is composed mainly by HOCl and do not rapidly lose chlorine. In this study, exposure of eight fungi at 12 mg/L ACC of NEW, effectively inhibited fungal growth at a concentration 4.5 times lower than acid EW at 54–56 mg/L (Buck et al., 2002).

Spore germination of *B. cinerea* (isolated from tomato) was inhibited 100% by NEW at 12 mg/L ACC and 3 min of treatment and by CO at 0.3 g/L. Comparable results were obtained with *near* NEW, where *B. cinerea* (fruit source not indicated) spore germination was inhibited 100% at 10 mg/L total residual chlorine and longer contact time (10 min) (Guentzel et al., 2010). In addition, Gómez Jaimes et al. (2017) reported that NEW at 18 mg/L ACC and 5 min contact time had 100% inhibition of spore germination of *B. cinerea* (isolated from blackberry) and no germ tube growth was observed. In another study, the inhibition of *B. cinerea* (isolated from strawberry) mycelial growth was evaluated with *near* NEW at 10, 25, 50, 100 and 200 mg/L ACC and 3-, 7- and 10-min contact time. It was reported that the ACC required to inhibit 100% mycelial growth was 50 mg/L ACC and 7 min contact time, while 10 mg/L ACC and 3 min inhibited 50% mycelial growth and a longer contact time (10 min) inhibited >90% mycelial growth (Guerra Sierra et al., 2019). These results show that NEW at 12 mg/L ACC may be an ecological novel alternative to CO to avoid produce loss and provide longer shelf life.

Spore germination of *C. gloeosporioides* and *C. siamense* (isolated from banana and papaya, respectively) was inhibited 100% by NEW at 12 mg/L ACC and 3 min of exposure and by CO at 0.3 g/L. Other studies show that NEW at 6 mg/L ACC and 5 min contact time had 100% inhibition of spore germination of *C. gloeosporioides* isolated from mango, guava and lychee, and no germ tube growth was observed (Gómez Jaimes et al., 2017).

Spore germination of *L. theobromae* (isolated from banana) was inhibited 100% by NEW at a concentration of 12 mg/L ACC and 3 min of treatment and by CO at 0.3 g/L. To our knowledge, this is the first study to evaluate the effectiveness of any type of electrolyzed water (acid, neutral or near neutral) against this fungus, thus resulting of great importance for the banana production industry offering a novel nontoxic treatment against this fungus.

Spore germination of *F. oxysporum* and *F. solani* (isolated from chili canario and chili habanero, respectively) was inhibited 100% by NEW at 12 mg/L ACC and 3 min of treatment. In contrast, these fungi were inhibited less than 25% by CO at 0.3 g/L. Similar results were observed with *F. solani* (isolated from chili and stevia) treated with NEW at 6 mg/L ACC and 5 min contact time (Gómez Jaimes et al., 2017). Audenaert et al. (2012) found that spore germination of *F. graminearum, F. culmorum, F. poae* and *F. sporotrichoides* (isolated from wheat) was inhibited 90–100% by NEW at 12 mg/L ACC after 4 h contact time. However, greater ACC (25, 100 and 200 mg/L) and greater contact time (48 h) completely inhibited spore germination. In addition, the reduction of fungal spore germination with NEW was generally higher than with the fungicides prothioconazole and fluoxastrobin (Audenaert et al., 2012). In another study, the inhibition of mycelial growth diameter of *Fusarium* sp. (isolated from pineapple) was evaluated with acid EW at 100, 200 and 300 mg/L ACC and 10 min contact time and 300 mg/L ACC was the most effective to suppress 35% of *Fusarium* sp. mycelia growth (Whangchai et al., 2017).

### 3.3 Effect of medium ACC of NEW and CO on spore germination of different fungi in pure culture

NEW at medium concentration (33 mg/L ACC) and 3-, 5- and 10-min contact time inhibited less than 50% of conidia germination of *A. niger*, however no conidia germination (inhibition of 100%) was observed at 53 mg/L ACC. In another study comparing acid EW with NEW at medium ACC (30 mg/L), NEW exposed reductions around 2–2.44 log_10_ conidia/mL while acid EW exposed reductions around 1.33–1.51 log_10_ conidia/mL against *A. flavus* (Xiong et al., 2010). However, both acid EW and NEW showed complete inhibition at 60 or 90 mg/L ACC treatments (high ACC) (Xiong et al., 2014). Similarly, another study comparing acid EW at medium ACC (39.4 mg/L ACC) with alkaline EW; alkaline EW showed no inhibition while acid EW showed complete inhibition of *A. parasiticus* fungal growth (Suzuki et al., 2002). On another research, enhancing NEW at 9 mg/L ACC (low ACC) with mild and hot temperature (50 °C or 70 °C), achieved 2 log_10_ CFU/mL reduction and complete inhibition of *A. flavus*, respectively. However, at 20 °C treatment temperature, medium ACC (33 mg/L) reduced 3.5 log_10_ CFU/mL and high ACC (174 mg/L) reduced 5.83 log_10_ CFU/mL of *A. flavus* (Yamaner et al., 2016).

### 3.4 Effect of high ACC of NEW and CO on spore germination of different fungi in pure culture

The highest concentration of NEW evaluated in this study (53 mg/L ACC) was the only treatment that inhibited 100% the spore germination of *A. niger* and *A. tamarii* (isolated from tomato and peach, respectively) among all other treatments. In contrast, these fungi were inhibited less than 25% by CO at 0.3 g/L. Similar to our findings, *A. flavus* and *A. niger* (isolated from various symptomatic plants) were eliminated by acid EW at 54 mg/L and 30 s contact time (Buck et al., 2002). Another research showed that no survivors of *A. flavus* (isolated from peanuts) were detected after either acid EW or NEW at 60 and 90 mg/L ACC treatments (Xiong et al., 2014). A recent investigation presented reductions of *A. flavus, A. nomius, A. parasiticus, A. carbonarius, A. niger, A. ochraceus*, and *A. westerdijkiae* around 1–1.9 log_10_ CFU/g with either acid EW at high ACC (60,85 and 121 mg/L) or with alkaline EW (Lemos et al., 2020). A possible variable that could lead to different results analyzed herein could be due to a difference in the *Aspergillus* strain. Nevertheless, our results are in harmony with previous authors which have shown that NEW is an effective treatment against the proliferation of *Aspergillus*.

NEW at any concentration and contact time and CO at 0.3 g/L inhibited less than 25% the fungus *R. stolonifer* (isolated from strawberry). In contrast to our results, 100% inhibition of spore germination and germ tube growth was observed with NEW at 5 mg/L ACC and 18 mg/L for *R. stolonifer* (isolated from soursop and yaca, respectively) with 5 min contact time (Gómez Jaimes et al., 2017). In another study, Guerra Sierra et al. (2019) reported that the concentration of near NEW required to inhibit 100% of mycelia growth of *R. stolonifer* (isolated from strawberry) was 10 mg/L ACC and 10 min contact time. Such differences may arise from *R. stolonifer* strains, which could be related with the isolation produce.

**Figure 3:**
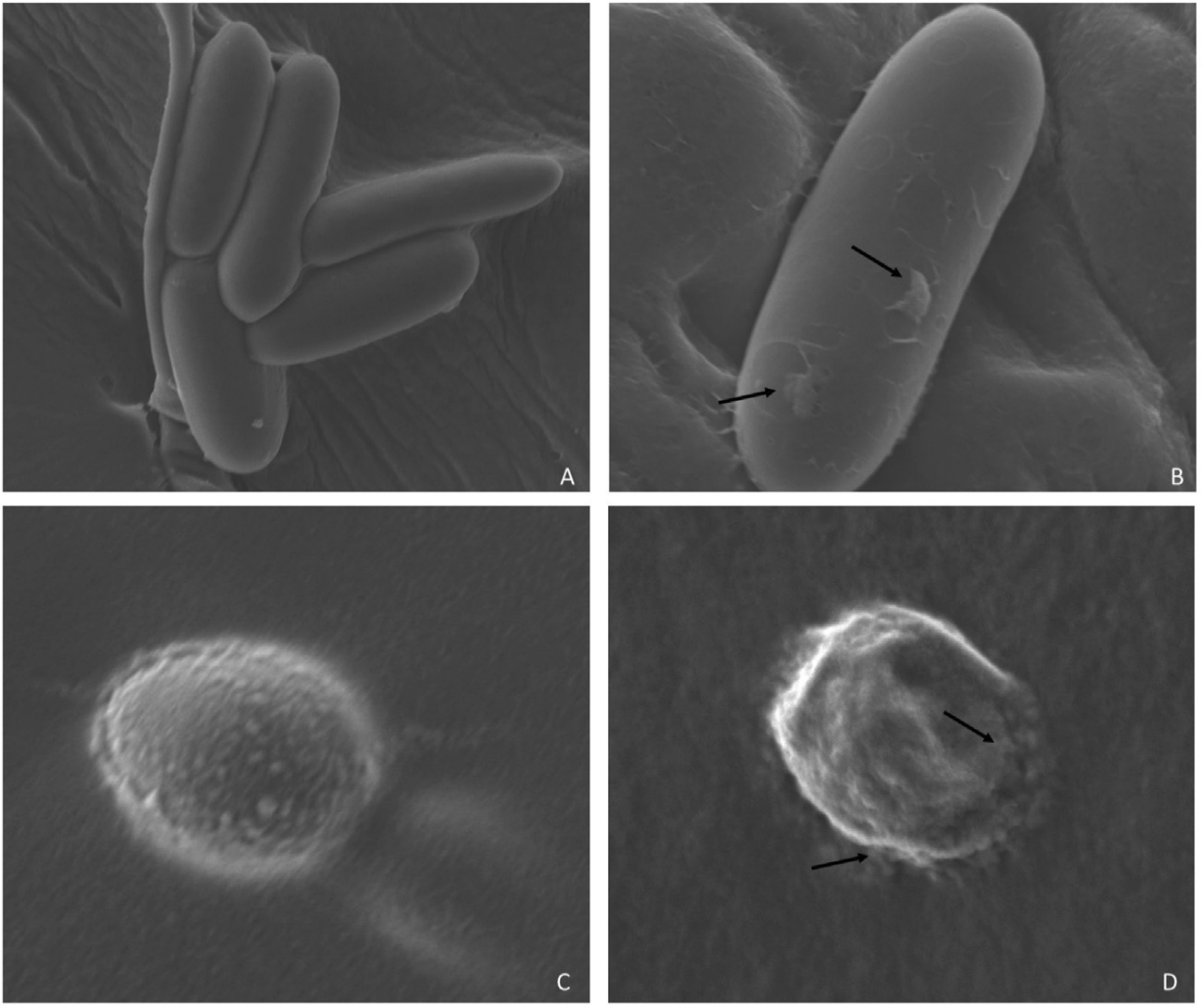
Scanning electron micrographs of *Colletotrichum gloeosporioides* (A: normal conidia; B: conidia treated with NEW at 12 mg/L ACC) and *Botrytis cinerea* (C: normal conidia; D: conidia treated with NEW at 12 mg/L ACC). The arrows show that the structures of cell wall shrank and partially cracked.

Chemical properties of EW, including the pH, ORP, and ACC have an effect on the antimicrobial efficacy of EW. Some authors indicate that the strong antimicrobial effect of EW is due to the pH (Rahman et al., 2010), others report that EW effectiveness is due to chlorine concentration (Xiong et al., 2014), and other authors indicate that EW effectiveness is due to the combination of the three factors (Hou et al., 2012). Differences in pH and ORP were not evaluated in this study, however, according to our results, NEW with greater concentrations of ACC were observed to exert a greater fungicidal effect. Buck et al. (2002) reported that the fungicidal capacity of EW is related with the thickness of the cell wall; thus, inhibition of thin cell wall spores treated with EW, such as *Botrytis* and *Monilinia*, can be accomplished in 30 s or less; while the inhibition of thick cell wall spores treated with EW, can be achieved in 2 min. In our study, NEW required higher ACC (53 mg/L), to inhibit the germination of *A. niger*, *A. tamarii* and *R. stolonifer* spores. These results may be related to the thickness of the cell wall of these fungi; as well as the content of intracellular chemical compounds, such as melanin, which constitutes a chemical defense mechanism. Furthermore, Xiong et al. (2014) reported that the target sites of EW should be the cell wall, and the membrane of the conidia and the mycelium generating irregular edges, swelling, contraction, partial cracking, chipping and cavities. According to these authors, ·OH is the most important fungicidal factor in NEW which can damage the normal morphological structure of conidia. These damages lead to the leakage of K^+^ and Mg^2+^ ions, consequently, cellular functions become abnormal. In our study, morphological changes due to EW effect were not documented, however, physical damage (irregular edges partial cracking and cavities) was observed in *C. gloeosporioides* with Scanning Electron Microscope JEOL IT-300LV (Figure 3).

## 4 Conclusions

Scientific research is driving towards the development of non-toxic products to avoid fruit decay during harvest, packaging and storage. This study contributed with new information on the effectiveness of NEW on several fungi spores isolated from tropical post-harvest fruits compared to a commonly used fungicide; copper oxychloride. NEW could be used as an alternative or supplement to conventional sanitizing practices, for general sanitation of greenhouses, harvesting materials, packing houses surfaces and for post-harvest fruit surfaces disinfection to prevent or manage fruit infection such as banana, chili, papaya, pineapple, peach and strawberry. The results presented herein can be used to understand the factors to consider when choosing the concentration and contact time of NEW on post-harvest fruits. Future research should be focused on *in vivo* effectiveness of NEW, which would complement this study and broaden the applications of this ecofriendly technology.

## Declarations

### Author contribution statement

Alfonso Vásquez-López: Conceived and designed the experiments; Performed the experiments; Analyzed and interpreted the data; Wrote the paper.

Rafael Gómez-Jaimes: Conceived and designed the experiments; Performed the experiments; Analyzed and interpreted the data.

Tania Villarreal-Barajas: Conceived and designed the experiments; Wrote the paper.

### Funding statement

This work was supported by the Consejo Nacional de Ciencia y Tecnología (10.13039/501100003141CONACYT), Mexico (grant number PROINNOVA-220362).

### Data availability statement

Data associated with this study has been deposited at *Alternaria alternata* MH560609 (501), *Aspergillus niger* HQ850370.1 (591), *Botrytis cinerea* MN589850 (569), *Cladosporium australiense* MN341230.1 (549), *Colletotrichum gloeosporioides* MH865167.1 (577), *Colletotrichum siamense* MT434615.1 (593), *Fusarium oxysporum* GU724514.1 (539), *Fusarium solani* KR708647.1 (550), *Lasiodiplodia theobromae* EU918707.1 (566), *Rhizopus stolonifer* AB113022.1 (852), *Aspergillus tamarii* MK638758.1 (1747).

### Declaration of interests statement

The authors declare no conflict of interest.

### Additional information

No additional information is available for this paper.
